# Influence of *Moringa oleifera* biodiesel–diesel–hexanol and biodiesel–diesel–ethanol blends on compression ignition engine performance, combustion and emission characteristics

**DOI:** 10.1039/c9ra09582a

**Published:** 2020-01-27

**Authors:** Selvakumar Ramalingam, N. V. Mahalakshmi

**Affiliations:** Department of Mechanical Engineering, Anna University Chennai Tamilnadu India rselva83@gmail.com +91 9790180177

## Abstract

In the current work, the influences of *Moringa oleifera* biodiesel–diesel–hexanol and *Moringa oleifera* biodiesel–diesel–ethanol blends on compression ignition engine characteristics were experimentally investigated. Experiments were conducted on a diesel engine at 0%, 25%, 50%, 75% and 100% load conditions run at a constant speed of 1500 rpm. The results revealed that B90-D5-H5 acquired the lowest BSFC and maximum BTE of 0.375 kg kW^−1^ h^−1^ and 28.8%, respectively, and B100 had the highest BSFC of 0.425 kg kW^−1^ h^−1^. B90-D5-H5 had the highest cylinder peak pressure of 74 bar at 4°CA aTDC. The maximum heat release rate (HRR) and longer ignition delay (ID) period of 44 J per °CA and 14.4°CA, respectively, were attained in the B90-D5-H5 blend. At 100% load condition, the lowest amount of carbon monoxide (CO) of 0.32% vol. was acquired in the B80-D5-E15 blend. The maximum nitric oxide (NO) emission of 1090 ppm was also acquired in the B80-D5-E15 blend. B100 had the lowest NO of 846 ppm; B80-D5-E15 had the lowest unburned hydrocarbon (UBHC) emission of 34 ppm at 100% load and the lowest smoke opacity of 34%. Biodiesel–diesel–alcohol blends improve engine performance and decrease emissions compared to the conventional diesel. The utilization of biodiesel–diesel–alcohol blends reduces the consumption of diesel. Hence, ethanol and hexanol are recommended as potential alternative additives in biodiesel–diesel blends to improve engine performance and reduce emissions.

## Abstract 2

In the current work, the influences of *Moringa oleifera* biodiesel–diesel–hexanol and *Moringa oleifera* biodiesel–diesel–ethanol blends on compression ignition engine characteristics were experimentally investigated.

## Graphical abstract

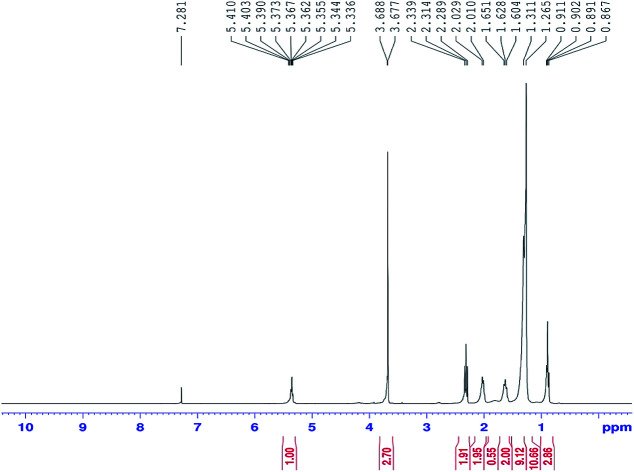

## 1. Introduction

Compression ignition engines play a vital role in the transportation, agricultural and power sectors, but these engines emit harmful emissions like carbon monoxide (CO), carbon dioxide (CO_2_), and oxides of nitrogen (NO_*x*_) when fueled by the conventional diesel. These emissions are the real contributors to global warming and environmental pollution and degradation.^1^ Moreover, increases in the cost of and demand for petroleum diesel related to the production and consumption of fossil fuels associated with the diminution of fossil fuel resources have led to the search for new alternative sources of renewable energy for diesel engines.^2^ Biodiesel is attaining a huge reputation as an environmentally friendly fuel and is one of the most predominant substitutes for conventional petroleum–diesel. Biodiesel is a long-chain fatty acid obtained from non-edible and edible sources, such as palm oil, sunflower oil, rapeseed oil, soybean oil, coconut oil, and groundnut oil as well as non-edible neem, rubber, jatropha, cotton, jojoba, castor, mahua, animal fats, waste frying oil, and micro-algae.^3^ In addition, the physico-chemical properties of methyl ester are almost similar to those of the conventional diesel.

The transesterification process is a general convenient and low-cost process to convert triglycerides into methyl ester, as noted by Mohammed Salaheldeen *et al.*^4^ and Hassan *et al.*^5^ In general, two types of catalysts are used, *i.e.*, homogeneous and heterogeneous catalysts. A heterogeneous catalyst is a minimal effect concentration catalyst as it reacts only on the uncovered dynamic surface. A homogeneous catalyst mixes into the reaction blend and allows an extreme interface with the catalyst and reactant molecules. Various homogeneous catalysts have been used in biodiesel production, such as NaOH, CH_3_ONa, KOH, and CH_3_OK as well as acid catalysts such as H_2_SO_4_, HCL, and HNO_3_, as observed by Elsanusi *et al.*,^2^ Mohammed Salaheldeen *et al.*^4^ and Tan *et al.*^6^

Although biodiesel has many advantages, it also has some disadvantages, such as its higher density and viscosity resulting in some problems during injection when used for a long period. Also, NO_*x*_ emission is another major problem when using biodiesel in diesel engines. After-treatment methods and recent technologies are used in reduction of NO_*x*_ emissions, but are limited in the commercial sector. Therefore, additional fuels are required to blend with biodiesel to improve the overall engine characteristics. Incidentally, alcohol has been tried as a supplementary fuel, as it supplies more oxygen and raises the heat of evaporation of the fuel, thus decreasing NO_*x*_ and particulate matter (PM) emissions. Several researchers have used two alcohols, like ethanol and methanol, in biodiesel syntheses. Ethanol can be derived from renewable resources and is mainly used in biodiesel production due to its lower cost and toxicity compared with other alcohols, as noted by Li D.-G.^7^ N. Yilmaz *et al.* (2012)^8^ used different additives like alcohols, metal additives, cetane improvers, and cold flow improvers to improve the fuel quality and combustion rate and decrease exhaust emissions.

Li *et al.*^7^ examined the effect of ethanol–diesel fuel blends on the performance and emission characteristics of a single-cylinder water-cooled direct injection diesel engine. The fuel blends were D95-E5 (95% diesel, 5% ethanol), D90-E10, D85-E15 and D80-E20. The results revealed that BSFC increased when ethanol percentage was increased because of its lower heating value. BTE improved when ethanol percentage was increased due to its lower boiling point compared with conventional diesel, as noticed by Abdullah *et al.*^9^ Huang *et al.*^11^ studied the influence of diesel–ethanol fuel blends on diesel combustion and emission characteristics. The experiment results exhibited lower CO and NO emissions for ethanol–diesel fuel blends compared to conventional diesel, but UBHC emission increased for biodiesel–ethanol fuel blends compared with neat diesel. Q. Fang *et al.* (2013)^10^ investigated the impact of ethanol–biodiesel–diesel fuel blends on four-cylinder diesel engine characteristics. The results showed that UBHC and CO emissions were raised, whereas NO_*x*_ emission decreased. The experimental results concluded that biodiesel with 20% ethanol decreased NO_*x*_ and smoke emissions. Hwanam *et al.*^12^ noticed that the addition of ethanol considerably enhances the combustion process, but its drawbacks are lower calorific value, cetane number, and flash and fire points, phase separation at lower temperatures and poor miscibility with conventional diesel. So, replacing conventional diesel with a perfect blend suitable for utilization in diesel engines is a difficult task.

Yao *et al.*^13^ noticed the disadvantages of lower alcohols, such as poor miscibility and blending solubility with diesel and phase separation at lower temperatures. Likewise, Moreau *et al.*^14^ observed that higher alcohols can be used as a resource due to their important thermodynamic and physical properties. Sivalakshmi *et al.*^15^ concluded that the higher alcohols with more carbon have a high cetane number and better miscibility with conventional diesel. Yesilyurt *et al.*^19^ investigated diesel engine characteristics fueled with biodiesel–diesel–1-butanol and biodiesel–diesel–*n*-pentanol blends at different loads and speeds. The studies reported that increases in alcohol percentage increased the BSFC and reduced the BTE. Alcohol blends obtained lower EGT, CO_2_ and NO emissions. 1-Butanol and *n*-pentanol can be used as alternative additives in biodiesel–diesel blends to improve performance and reduce emissions. Yesilyurt *et al.^19^* also studied the effect of injection pressure on diesel engine characteristics fuelled with waste frying oil biodiesel and diesel blends. The CO, UBHC, smoke, torque and brake power reduction in biodiesel blends were comparable to conventional diesel, whereas the EGT, NO and CO_2_ increased for biodiesel. Nour *et al.^20^* carried out research on a diesel engine fuelled with biodiesel–higher alcohol blends. The study reported that biodiesel–higher alcohol blends had high stability with no phase separation. NO and smoke emissions were reduced, but CO and UBHC emissions increased for biodiesel–higher alcohol blends. Ashok *et al.^21^* investigated the effect of biodiesel–*n*-pentanol blends on diesel engine characteristics. Addition of *n*-pentanol to biodiesel improved thermal efficiency and reduced UBHC and CO emission by about 15–43% and 33–50%, respectively, compared to pure biodiesel. Addition of *n*-pentanol above 40% had a negative effect on diesel engine performance characteristics.

The above literature studies noted the significance of diesel engine emission reduction to protect the environment. Also, concerns about environmental pollution, global warming, ozone depletion, depletion of fossil fuel sources and stringent emissions standards have provoked research curiosity to search for new alternatives for diesel engines. In this present work, biodiesel was synthesized from *Moringa oleifera* oil. Huge amounts of unpicked *Moringa oleifera* seeds are available in the trees even after harvesting. Therefore, collected waste seeds can be used as a potential feedstock for biodiesel production due to their availability and low cost. Without diesel engine modification, fueling with pure biodiesel creates more emissions due to its inferior properties, like higher viscosity and density, which affect atomization and evaporation, resulting in lower brake thermal efficiency. Higher alcohols blended with biodiesel and diesel have improved the fuel properties and enhanced combustion efficiency through proper combustion, as seen in the literature. However, minimal research work has been carried out to investigate the diesel engine performance and combustion characteristics when fueled with *Moringa* biodiesel–diesel–ethanol and *Moringa* biodiesel–diesel–hexanol blends without engine modification. The addition of a lower alcohol to biodiesel reduces the viscosity and density, cetane number, and calorific value and causes poor stability and phase separation at lower temperatures. The higher alcohol was able to overcome the drawbacks of lower alcohols with a higher cetane number and heating value, better miscibility with diesel, and stability and no phase separation at higher temperature. Hence, in the current study, an attempt has been made to evaluate the effects of biodiesel–diesel–ethanol and biodiesel–diesel–hexanol blends on diesel engine performance and combustion characteristics.

## 2. Materials and methods

### 2.1 Synthesis of biodiesel characterization

The biodiesel experiment was carried out using the design matrix given in Table 1. Initially, 100 g of raw oil was transferred into a glass round bottom flask and heated using an electrical heater. The methanol and sodium methoxide are preliminarily mixed by magnetic stirrer. The mixture was poured into a three-necked flask and stirred at certain reaction conditions. After the reaction, the solution was shifted into a conical separating funnel for separation. The separated biodiesel was washed with heated distilled water and heated to 100 °C to remove excess methanol, moisture content, non-reacted catalyst and glycerol. The excess methanol affects the biodiesel properties. Among all the trials, the highest yield of 96.71% was acquired with the following reaction conditions: molar ratio 6 : 1, reaction temperature 55 °C, catalyst 1.8 wt%, reaction time 120 min and stirrer speed 600 rpm. The physicochemical properties of raw oil, biodiesel and diesel are depicted in Table 2.

**Table 1:** List of compounds in biodiesel

Name of the component	Molecular formula	Molecular weight (g mol^−1^)	Retention time (min)	Peak area (%)
Heptanoic acid, methyl ester	C_8_H_16_O_2_	144	6.5	0.0073
Octanoic acid, methyl ester	C_9_H_18_O_2_	158	8.73	0.182
Nonanoic acid, methyl ester	C_10_H_20_O_2_	172	10.99	0.0021
Decanoic acid, methyl ester	C_11_H_22_O_2_	186	13.39	1.1664
Undecanoic acid, methyl ester	C_12_H_24_O_2_	200	15.47	0.0098
Nonanoic acid, 9-oxo-, methyl ester	C_10_H_18_O_3_	186	15.84	0.0159
Dodecanoic acid, methyl ester	C_13_H_26_O_2_	214	18.87	22.3309
Tridecanoic acid, methyl ester	C_14_H_28_O_2_	228	20.97	0.0345
Pentadecanoic acid, methyl ester	C_16_H_32_O_2_	256	26.4	0.0238
9-Hexadecenoic acid, methyl ester, (Z)-	C_17_H_32_O_2_	268	28.59	1.2212
Hexadecanoic acid, methyl ester	C_17_H_34_O_2_	270	29.54	12.76
Cyclopropaneoctanoic acid, 2-hexyl-, methyl ester	C_18_H_34_O_2_	282	30.91	0.122
Hexadecanoic acid, 15-hydroxy-, methyl ester	C_17_H_34_O_3_	286	31.59	0.1977
9-Octadecenoic acid, methyl ester	C_19_H_36_O_2_	296	33.79	27.6555
Heptadecanoic acid, 10-methyl-, methyl ester	C_19_H_38_O_2_	298	35.08	4.037
7,10-Octadecadienoic acid, methyl ester	C_19_H_34_O_2_	294	35.67	0.0827
10-Nonadecenoic acid, methyl ester	C_20_H_38_O_2_	310	35.81	0.1073
Nonadecanoic acid, methyl ester	C_20_H_40_O_2_	312	36.24	0.0402
11-Eicosenoic acid, methyl ester	C_21_H_40_O_2_	324	37.96	4.6435
Eicosanoic acid, methyl ester	C_21_H_42_O_2_	326	38.66	7.4426
Heneicosanoic acid, methyl ester	C_22_H_44_O_2_	340	40.3	0.0405
13-Docosenoic acid, methyl ester	C_23_H_44_O_2_	352	41.83	0.161
Octadecanoic acid, 5,9,13,17-tetramethyl-, methyl ester,	C_23_H_46_O_2_	354	42.76	0.68
Tricosanoic acid, methyl ester	C_24_H_48_O_2_	368	44.32	0.1533
Tetracosanoic acid, methyl ester	C_25_H_50_O_2_	382	47.06	2.118

**Table 2:** Physical and chemical properties of diesel, MO oil and MOME

Properties	Diesel ASTM D975	MO oil	MOME ASTM D6751	Test method
Kinematic viscosity @ 40 °C (mm^2^ s^−1^)	2.389	16.78CST	4.97CST	ASTM D445
Density (kg m^−3^)	850	927	867.7	ASTM D1298
Specific gravity	0.85	0.977	0.867	—
Flash point (°C)	51	314	156	ASTM D93
Fire point (°C)	53	322	162	ASTM D93
Cloud point (°C)	0	17	0	ASTM D2500
Pour point (°C)	−15	11	−5	ASTM D97
Carbon (wt%)	85.26	76.08	78.54	ASTM D5291
Oxygen (wt%)	0	12.63	13.4	ASTM D5291
Hydrogen (wt%)	14.36	12.52	11.02	ASTM D5291
Sulfur content (wt%)	0.159	0.308	0.005	ASTM D5453
Nitrogen content (wt%)	0.293	1.0852	0.385	ASTM D5291
Higher calorific value (MJ kg^−1^)	43.087	38.977	39.54	ASTM D240
Copper strip corrosion (class 1)	Class 1a	Class 1b	Class 1a	ASTM D130
Carbon residue (% mass)	0.0109	0.0202	0.013	ASTM D4530
Acid value (mg KOH per g oil)	0	10.36	0.67	ASTM D664
Saponification value (mg KOH per g·oil)	0	232.3	92.34	ASTM D5558
Iodine value (mg iodine oer 100 g·oil)	—	42.87	17.23	ASTM D5554
Cetane index	56	57	65	ASTM D976

### 2.2 Characterization of methyl ester

**Fig. 1:**
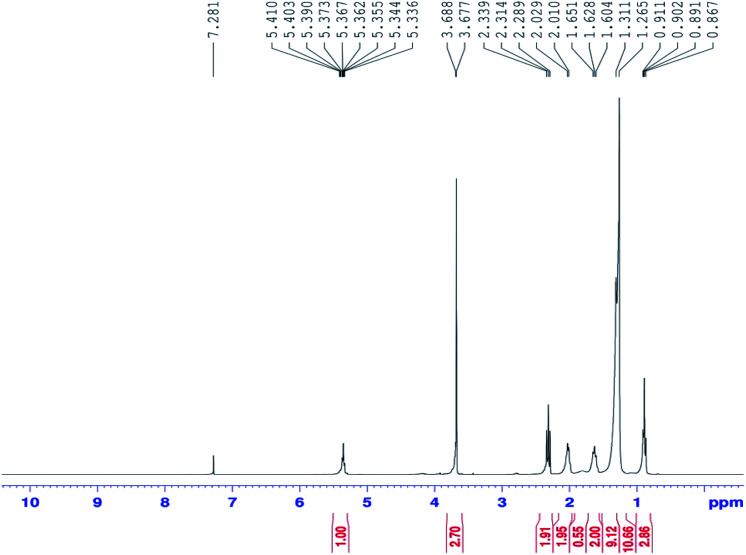
^1^H NMR spectrum of biodiesel.

**Fig. 2:**
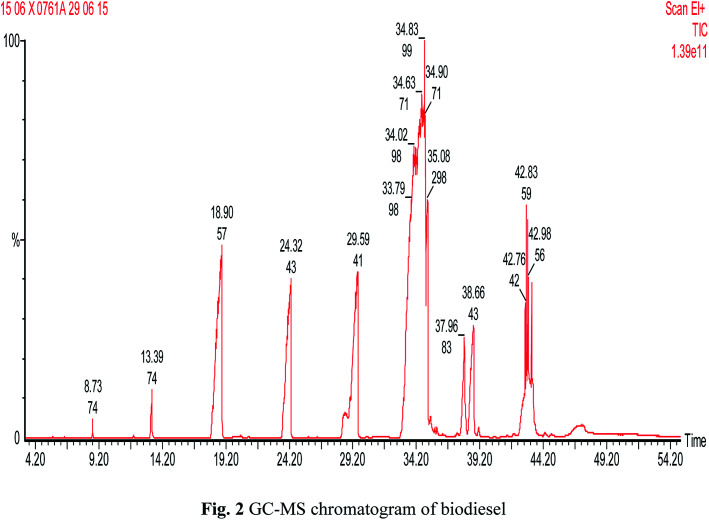
GC-MS chromatogram of biodiesel.

Fig. 1 shows the proton nuclear magnetic resonance (^1^H NMR) spectrum for biodiesel (Bruker Nonobay, 400 MHz). From Fig. 1, the peak at 3.6 δ ppm shows the presence of methoxy protons and the peak at 2.3 δ ppm confirms the presence of alpha methylene proton. These two peaks are sufficient to confirm the presence of methyl ester. Methyl ester conversion rate or percentage will also be measured through ^1^H NMR analysis. In contrast, the above peaks at 3.6 δ ppm and 2.3 δ ppm were not identified for either diesel or raw *Moringa oleifera* oil. Fig. 2 shows the GC-MS chromatogram (PerkinElmer Clarus 500; software: TurboMass ver. 5.2.0) of the biodiesel used to confirm the composition of oil and ester. In the GC-MS chromatogram, a capillary column (Elite-5MS; 95% dimethyl polysiloxane, 5% phenyl) was used. The length of the column is 30 m with an inner diameter of 250 μm. The initial temperature was 60 °C; it was increased to 150 °C and 280 °C with flow rates of 6 m^3^ s^−1^ and 4 m^3^ s^−1^ and holding times of 2 and 5 min, respectively. The injector temperature was 280 °C and helium was used as the carrier gas at a flow rate of 1 ml min^−1^ with a split ratio of 1 : 10. Mass spectrometer conditions were as follows: mass range of 40–600 amu and electron ionization with 70 eV of electron energy. The overall running time was around 55 min. The CG/MS test confirmed the proportion of unsaturated and saturated fatty acid components, as seen in Table 1. Increased presence of saturated fatty acids improved biodiesel properties such as cetane number, pour point, density, flash point, and oxidation stability.

## 3. Experimental setup and methodology

The experiment was carried out on a single-cylinder air-cooled diesel engine with rated power of 4.4 kW. The standard injection pressure and injection time were 210 bar and 23° TDC, respectively. A standard burette, orifice meter, and K type thermocouples were engaged to measure the fuel flow rate, airflow rate, and different temperatures, respectively. For loading, an electrical dynamometer was used. It comprises a DC generator and a load bank. The electrical load was varied from 0 to 16 amps. The engine emissions, like UBHC, CO, NO, O_2_ and CO_2_, were determined using a HORIBA MEXA-584L gas analyzer. The analyzer specifications are shown in Table 3. The smoke emission was measured by an AVL smoke meter. The engine in-cylinder pressure was measured by a Kistler 7613C quartz transducer with a pressure range of 0 to 250 bar.

**Table 3:** Emission analyzer specifications

Make	HORIBA MEXA-584L
Power supply	100 V to 240 V AC, 50/60 Hz
Warm up time	5 minutes
CO	0.00% to 10.00% vol.
HC	0.00 to 10 000 ppm vol.
CO_2_	0.00% to 20.00% vol.
Air fuel ratio (AFR)	10.0 to 30.0
Lambda	0.000 to 9.999
O_2_	0.00% to 25.00% vol.
NO	0 to 5000 ppm vol.
Engine rpm	0 to 9990 rpm
Temperature range	0 to 150 °C

**Fig. 3:**
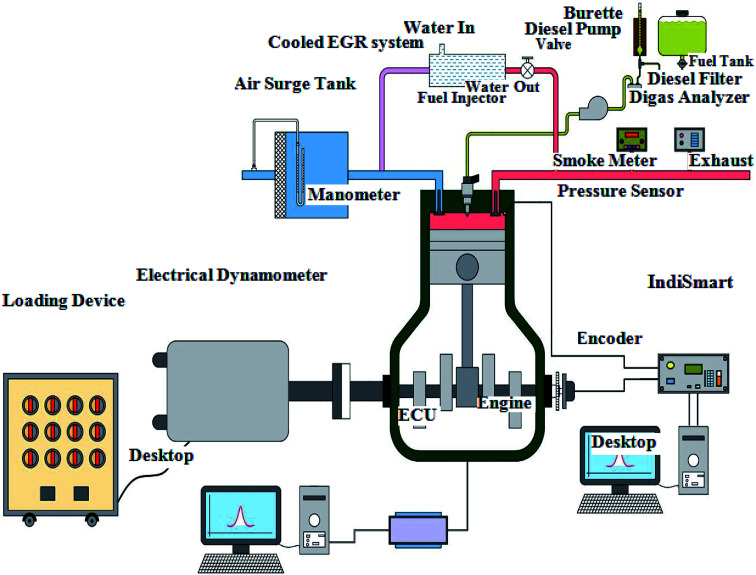
Schematic diagram of experimental layout.

The signals were received by a data acquisition system (PCIMIO 16-1 DAQ, National Instrument) and recorded as individual crank angle data from the crank angle encoder (Autonics rotary incremental ES50S8-360-3-T-1). The crank angle encoder specifications are depicted in Table 4. The analog signal was converted to digital through an analog-to-digital converter (ADC). The encoder gives one pulse for each degree to get 360 pulses for each revolution for crank angle mapping. The LabView software was programmed by National Instrument (NI) for real-time data analysis and recorded in-cylinder pressure data parallel to crank angles. The engine characteristics were recorded at 0%, 25%, 50%, 75% and 100% load conditions for biodiesel–diesel blends. The engine and dynamometer specifications are shown in Table 5. The schematic depiction of the test engine is illustrated in Fig. 3. The experiments were carried out three times for repeatability and average values were used for the calculations.

**Table 4:** Rotary encoder specifications

Make	Autonics (ES50S8-360-3-T-1), incremental
Power supply	5 V DC
Resolution	1 ppr (pulse per revolution)
Max. response frequency	300 Hz
Current consumption	Max. 80 Ma
Insulation resistance	Min. 100 MΩ
Dielectric strength	750 V AC 50/60 Hz for 1 minute
Starting torque	Max. 0.0007 nm
Moment of inertia	Max. 80 g cm^2^
Shaft loading	Radial: max. 10 kg F; thrust: max. 2.5 kg F
Max. speed	5000 rpm

**Table 5:** Test engine specifications

Make	Kirloskar
Model	TAF 1, 4 stroke, DI injection
Cooling system	Air
Displacement	661 cc
Bore	87.5 mm
Stroke	110 mm
Compression ratio	17.5 : 1
Combustion chamber	Hemispherical bowl in piston
Rated speed	1500 rpm
Power	4.4 kW
Injection mode	Mechanical
Nozzle opening pressure	210 bar
Number of orifices	3
Fuel injection timing	23° bTDC
Specific fuel consumption	251 g kW^−1^ h^−1^
IVO	15° bTDC
IVC	33° aBDC
EVO	30° bBDC
EVC	14° aTDC

## 4. Uncertainty analysis for diesel engine performance parameters

Uncertainty analysis is essential to check the accuracy of experiments. The accuracies of fuel measurement, speed, time, and brake power tests were ±1.1%, ±10 rpm, ±0.15 s and ±0.042 kW, respectively. The uncertainties of BSFC, BP, TFC, BSEC, and BTE were calculated by the root-sum-square measurement method.^18^ The specified engine performance for total and percentage uncertainties limits is given in Table 6.1
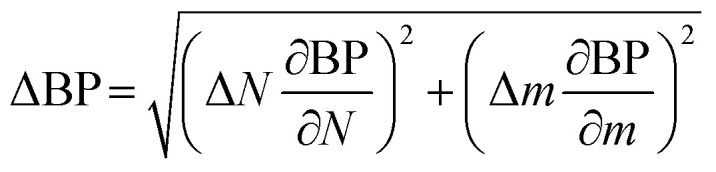
2
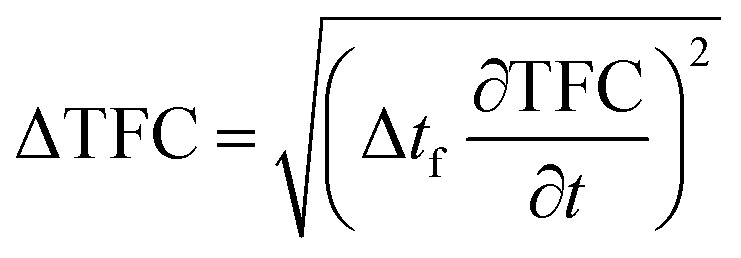
3
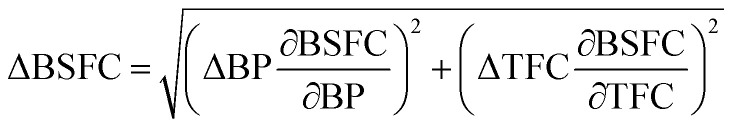
4
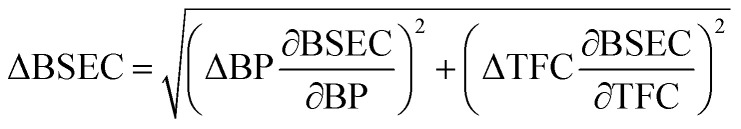
5
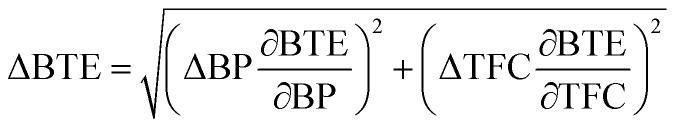


**Table 6:** Uncertainties for engine performance

S. no.	Engine performance characteristics	Total uncertainties	Percentage uncertainties
1	TFC	±0.0076 kg kW^−1^ h^−1^	±0.703%
2	BP	±0.042 kW	±1.10%
3	BSFC	±0.00365 kg kW^−1^ h^−1^	±1.30%
4	BSEC	±0.153 MJ kW^−1^ h^−1^	±1.29%
5	BTE	±0.396%	±1.29%

## 5. Results and discussion

### 5.1 Performance characteristics

**Fig. 4:**
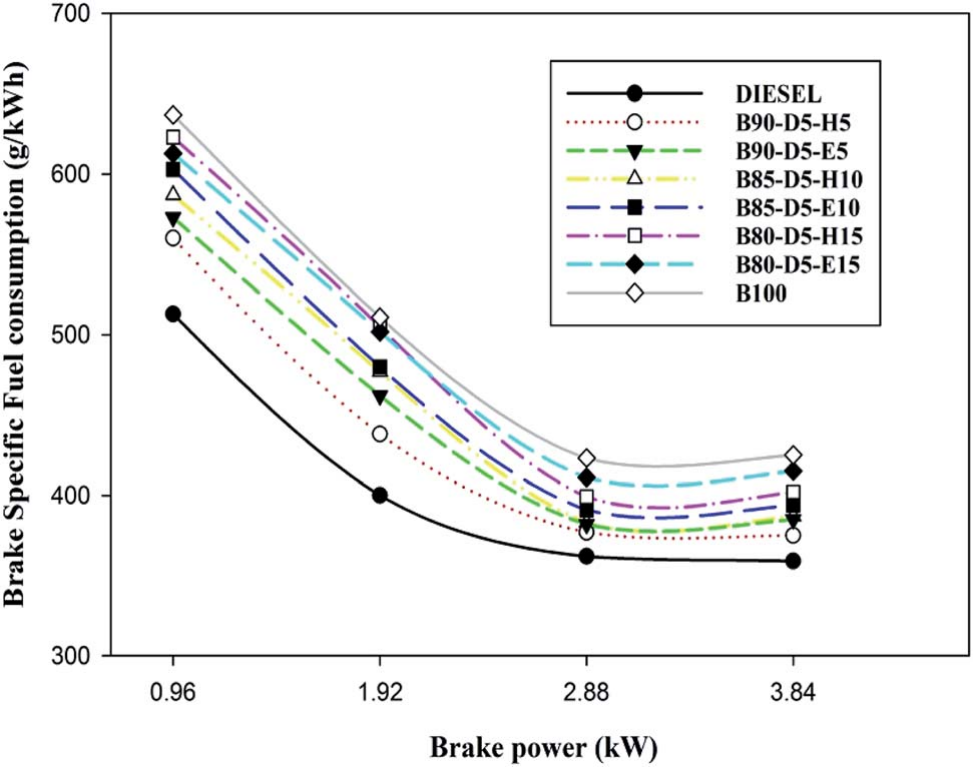
Variation of BSFC with brake power for diesel–biodiesel–alcohol blends.

Engine performance is a significant factor for calculating fuel economy; it is represented by BTE and BSFC. Fig. 4 represents brake specific fuel consumption (BSFC) relative to BP for biodiesel–diesel–alcohol blends. It is noted that the B90-D5-H5 blend acquired the lowest BSFC of 0.375 kg kW^−1^ h^−1^, which is 5% greater than conventional diesel, 12% less than the B100 and 10% less than the B80-D5-E15. B100 has the maximum BSFC compared with biodiesel–diesel–alcohol blends and conventional diesel. This is because of its inferior fuel properties, such as high viscosity and lower calorific value, compared with biodiesel–diesel–alcohol blends. High viscosity causes poor atomization and vaporization of fuel, causing partial combustion resulting in higher BSFC and lower BTE. Further, biodiesel–diesel–hexanol blends have better performances compared with biodiesel–diesel–ethanol blends. This is due to their enhanced fuel properties, like high heating value and high cetane number. Also, the diesel engine consumed more biodiesel fuel to obtain similar engine power output to diesel due to poor combustion efficiency. A similar trend was observed by Babu and Anand.^16^

**Fig. 5:**
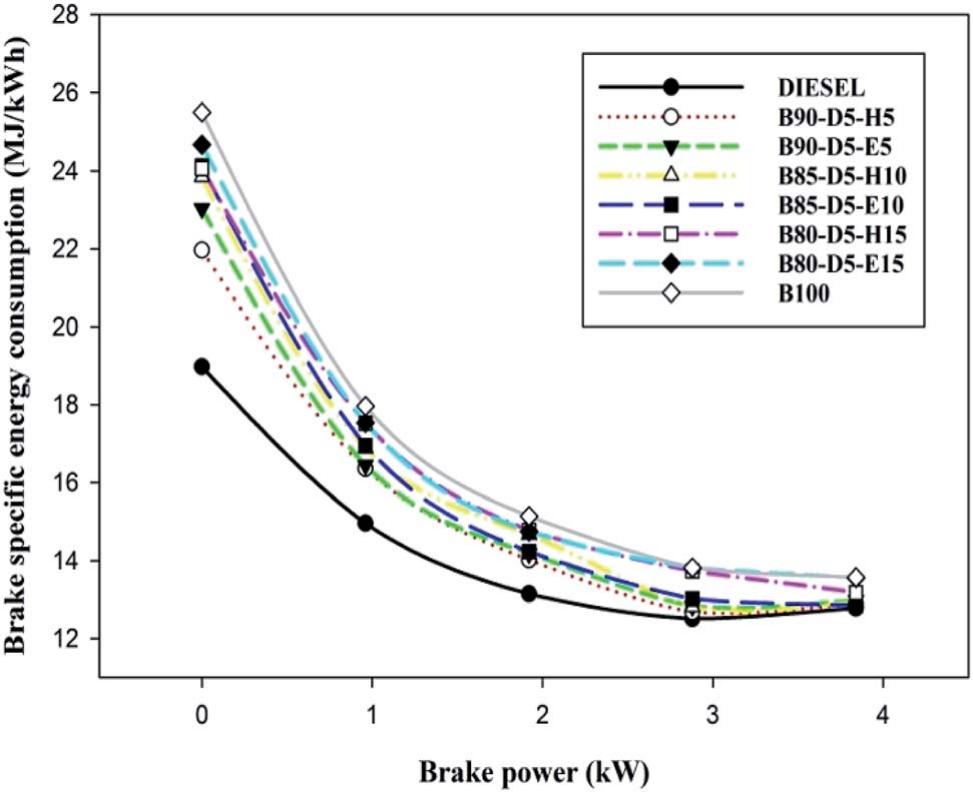
Variation of BSEC with brake power for diesel–biodiesel–alcohol blends.

Fig. 5 illustrates the difference of brake specific energy consumption (BSEC) *versus* BP for various biodiesel–diesel–alcohol blends, such as diesel, B100, B90-D5-H5, B90-D5-E5, D85-D5-H10, B85-D5-E10, B80-D5-H15 and B80-D5-E15. The lowest BSEC of 12.89 MJ kW^−1^ h^−1^ was acquired from B90-D5-H5, which is 2% less than biodiesel and 2% greater than conventional diesel. This is due to the biodiesel–diesel–hexanol blend having higher heating value with proper fuel–air mixture caused by complete combustion resulting in lower BSEC with higher power output. The B80-D5-E15 blend obtained a maximum BSEC of 13.56 MJ kW^−1^ h^−1^ at 100% load, which is 4% better than neat diesel. A similar trend was noticed by El-Seesy *et al.*^17^ Neat diesel has the lowest BSEC compared with biodiesel–diesel–alcohol fuel blends owing to its higher calorific value with lower viscosity, which leads to better combustion.

**Fig. 6:**
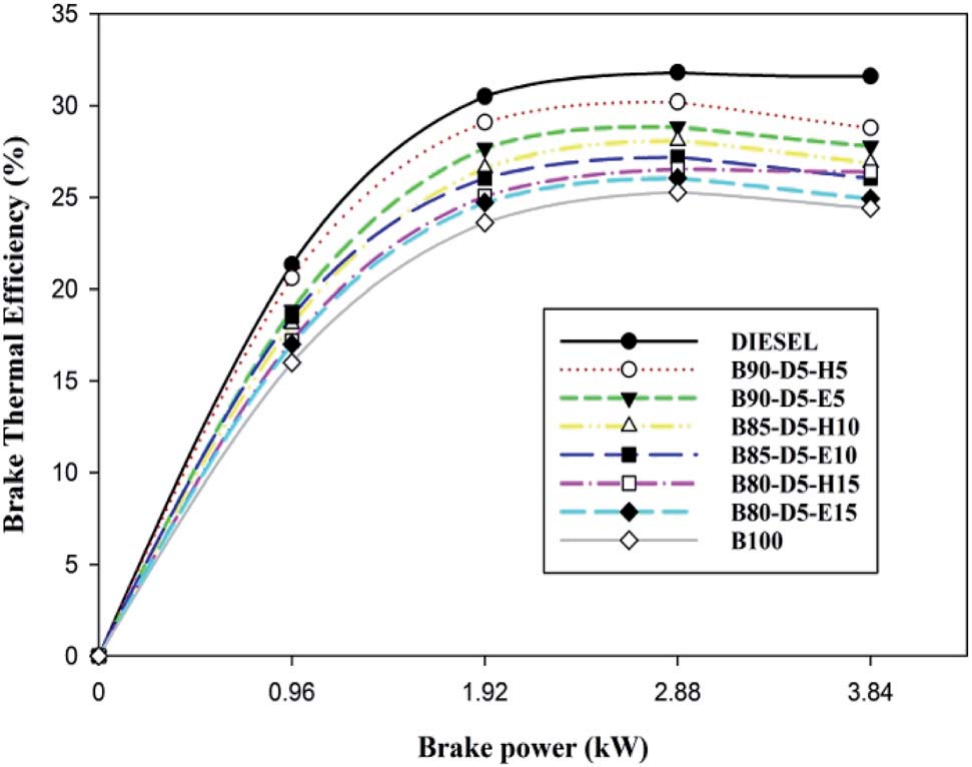
Variation of BTE with brake power for diesel–biodiesel–alcohol blends.

Fig. 6 illustrates the variation of brake thermal efficiency (BTE) with BP for biodiesel–diesel–alcohol fuel blends. Fig. 6 reveals that BTE rose with a rise in engine load and decreased with increasing percentage of biodiesel–alcohol blends in neat diesel. This is due to the addition of more alcohol or more percentage of biodiesel content into diesel reducing the calorific value of the blends and causing more consumption of fuel with minimum BTE compared to neat diesel. Also notice that BTE increased with an increase in engine load from zero to full load, due to more fuel burning in the combustion releasing more heat, resulting in in-cylinder pressure with raised temperature. The B90-D5-H5 fuel blend acquired the highest BTE of 28.8%, which is 2.2% less than neat diesel, 4.2% greater than the B80-D5-E15 and 4.4% greater than pure biodiesel. At all operating conditions, B100 has the least BTE compared with other blends due to its higher density and viscosity, which damaged the fuel spray characteristics of the tested fuels, resulting in partial combustion and lower brake thermal efficiency.

### 5.2 Combustion characteristics

**Fig. 7:**
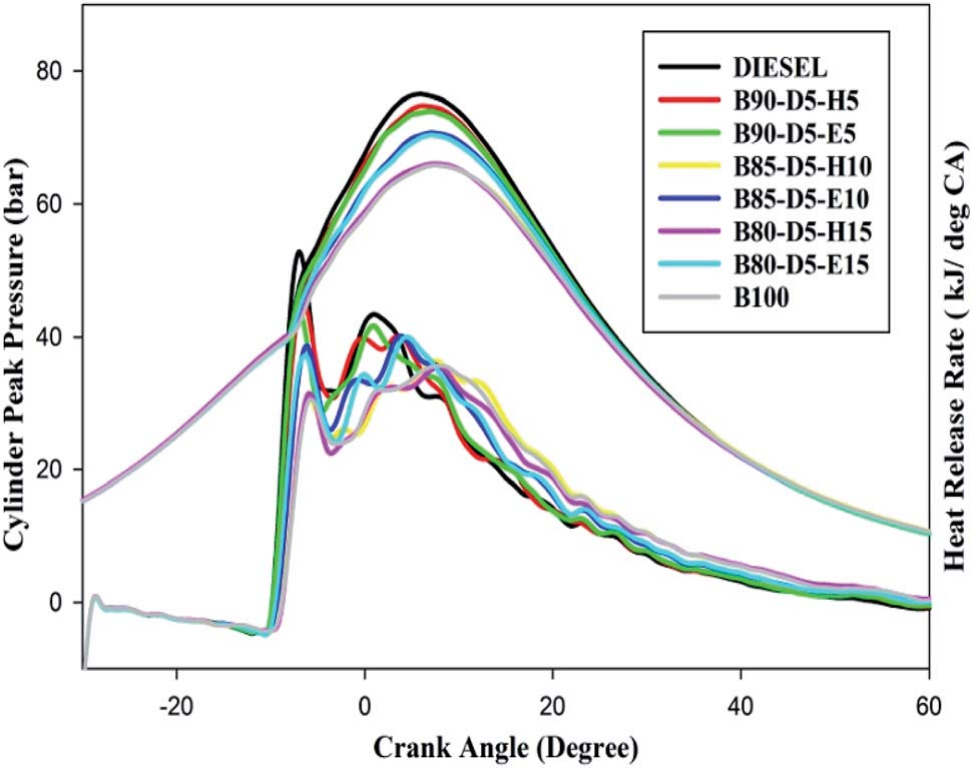
Variation of CPP and HRR with crank angle for diesel–biodiesel–alcohol blends.

Fig. 7 depicts the variation of in-cylinder peak pressure (CPP) against crank angle for different biodiesel–diesel–alcohol blends. From Fig. 7, it can be seen that CPP rises with a rise in engine load for biodiesel–diesel–alcohol fuel blends. However, CPP decreased with the increases in biodiesel percentage in diesel blends. This is because adding more alcohol to biodiesel–diesel blends suppressed the in-cylinder pressure and temperature caused by the cooling effect, resulting in lower CPP. Moreover, at minimum load, the CPP was minimized due to lower in-cylinder pressure with temperature, which reduced the evaporation rate of the fuels and resulted in partial combustion and lower CPP. Furthermore, pure biodiesel achieved minimum CPP compared to other tested fuels due to its poor volatility and higher viscosity reducing the evaporation rate, resulting in a minimum amount of fuel burned in the premixed combustion compared to diesel and biodiesel–diesel–alcohol blends, which leads to lower CPP. The B90-D5-H5 obtained the maximum CPP of 74 bar, 2 bar less than neat diesel, 4 bar greater than B80-D5-E15 and 9 bar higher than pure biodiesel. At all operating conditions, biodiesel–diesel–alcohol blends showed lower CPP compared with diesel. This is due to the addition of more biodiesel–alcohol to neat biodiesel reducing the heating value of the blends; also, increases in alcohol percentage increased the specific heat of the blends, resulting in more heat absorbed during evaporation, which suppressed the in-cylinder temperature and pressure. The B80-D5-H15 and B80-D5-E15 blends showed minimum CPP compared with diesel.

Variation of heat release rate (HRR) against crank angle for different biodiesel–diesel–alcohol blends at full load condition is also illustrated in Fig. 7. The HRR is mainly affected by fuel properties like calorific value, burning velocity, in-cylinder temperature and pressure, volatility of fuel, latent heat of evaporation, and fuel injection pressure and timing. Additionally, it is influenced by the amount of fuel burned in the premixed zone rather than controlled combustion and late combustion. B90-D5-H5 has the highest HRR of 44 MJ per °CA compared to other tested fuels, which is 8 MJ per °CA lower than conventional diesel, 9 MJ per °CA higher than pure biodiesel and 7 MJ per °CA higher than the B80-D5-E15 blend. B90-D5-H5 has maximum HRR because of its greater heating value with higher cetane number. The higher cetane number improves the evaporation rate of the tested fuels and results in higher HRR. The presence of excess oxygen and a proper fuel–air mixture cause complete combustion and result in higher HRR for biodiesel–diesel–alcohol blends. Biodiesel–diesel–ethanol blends have lower HRR compared with biodiesel–diesel–hexanol blends because of their lower heating value, which reduces the HRR. Addition of more alcohol in biodiesel–diesel blends reduced the HRR of biodiesel–diesel–alcohol blends by increasing the specific heat of the testing fuel, which leads to more heat absorbed during evaporation and results in reduced in-cylinder temperature and pressure.

**Fig. 8:**
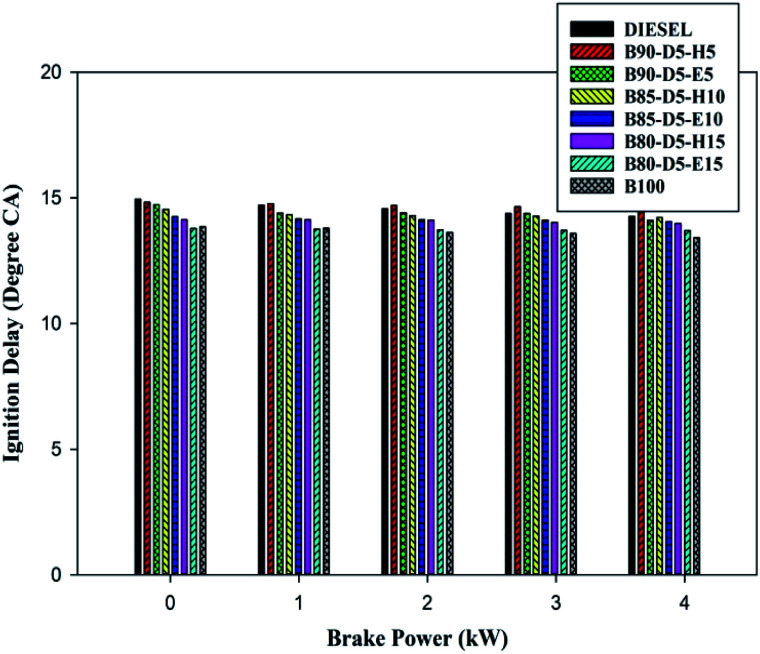
Variation of ignition delay with brake power for diesel–biodiesel–alcohol blends.

The physicochemical properties seriously influenced the ignition delay period of the testing fuels, especially viscosity, density, lower cetane number and lower in-cylinder properties. The delay period occurred between SOI and SOC. The variation of ID period *versus* BP for biodiesel–diesel–alcohol fuel blends is depicted in Fig. 8. From Fig. 8, it can be seen that at minimum load, the delay period was higher for all tested fuels, whereas when raising the engine load from 0% to 100%, the ID period decreased for all fuels. This is because rises in in-cylinder pressure and temperature, which enhanced the vaporization rate of testing fuels, resulted in a shorter delay period. The conventional diesel, B90-D5-H5, B90-D5-E5, B80-D5-H15, B80-D5-E15, and biodiesel have ID periods of 14.2 °CA, 14.45 °CA, 14.21 °CA, 13.7 °CA, 13.5 °CA, and 13.2 °CA, respectively. Biodiesel–diesel–alcohol fuel blends have the lowest ID periods because of their high cetane numbers, which reduce the ID period with a better evaporation rate.

### 5.3 Diesel engine emission characteristics

**Fig. 9:**
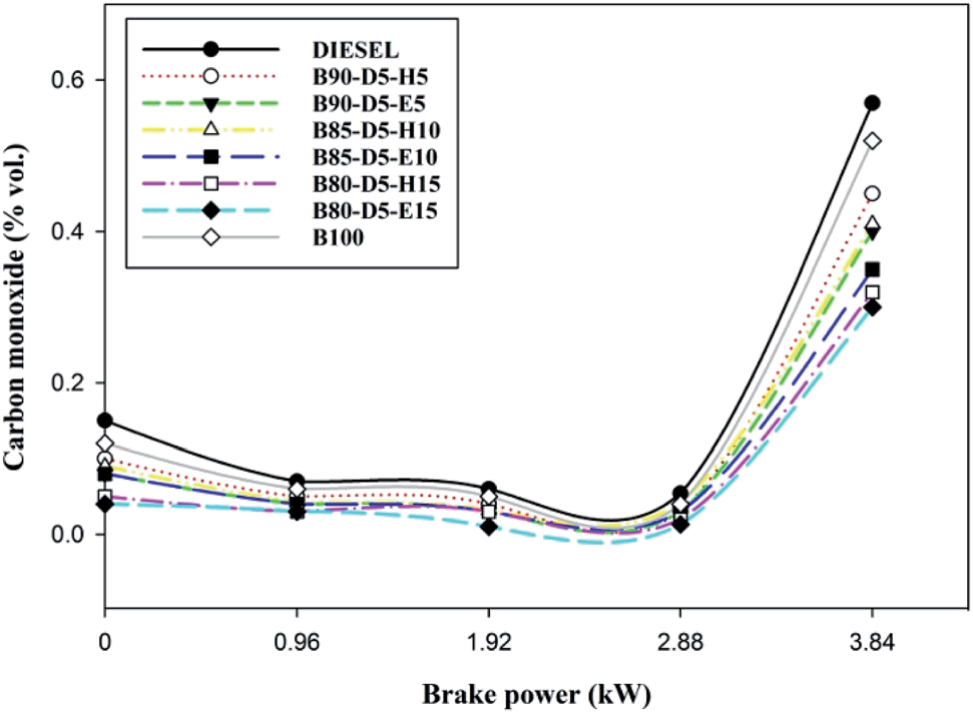
Variation of CO emission with brake power for diesel–biodiesel–alcohol blends.

Variation of CO emission *versus* BP for biodiesel–diesel–alcohol blends is depicted in Fig. 9, revealing that the CO rose with a rise in engine load from no load to 100% load for all fuels. At no load, CO emission was slightly higher because of lower in-cylinder pressure and temperature, which decreased the evaporation rate of the fuels and resulted in incomplete combustion with more CO formation. However, at full load, CO emission was maximum due to a deficiency of oxygen, lower ignition delay period, and the higher quantity of fuels causing incomplete combustion with higher CO emission formation. B80-D5-E15 has a minimum CO emission of 0.32% vol., which is 0.25% vol. less than conventional diesel, 0.02% vol. lower than pure biodiesel and 0.20% vol. lower than the B90-D5-H5. The CO is lower for biodiesel–diesel–ethanol blends compared with biodiesel–diesel–hexanol and conventional diesel. This is because ethanol has lower viscosity and lower latent heat of evaporation compared to biodiesel–diesel–hexanol fuel blends. Also, lower viscosity enhanced the atomization process and evaporation rate, resulting in incomplete combustion. Further, the molecular structure of biodiesel and oxygen content in alcohol fuel causes complete combustion.

**Fig. 10:**
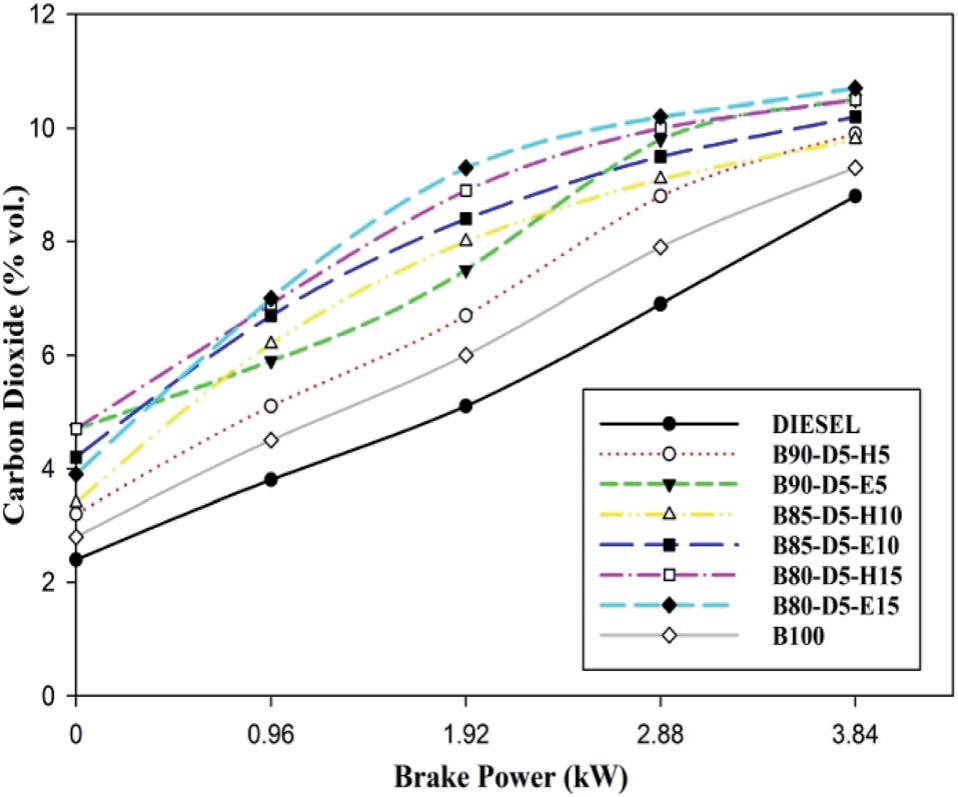
Variation of CO_2_ emission with brake power for diesel–biodiesel–alcohol blends.

Fig. 10 illustrates the variation of carbon dioxide (CO_2_) against BP for different biodiesel–diesel–alcohol blends. Fig. 10 reveals that CO_2_ rose with a rise in engine load for all biodiesel–diesel blends due to the occurrence of oxygen enclosure in alcohol fuel blends and higher in-cylinder temperature and pressure enhancing the combustion process. The CO_2_ emission is higher for biodiesel–diesel–ethanol blends compared with biodiesel–diesel–hexanol blends and conventional diesel. This is because of the lower viscosity and presence of oxygen, which enhanced the vaporization rate of the tested fuels, resulting in incomplete combustion with higher carbon dioxide emission formation. B80-D5-E15 has a maximum CO_2_ emission of 10.5% vol., which is 2.4% vol. greater than diesel, similar to pure biodiesel and 1.2% vol. higher than the B80-D5-E15. Higher alcohol blends have lower carbon dioxide emission because higher latent heat and higher viscosity reduce the evaporation rate by absorbing more heat during evaporation, resulting in reduced in-cylinder temperature and pressure that leads to partial combustion and lower CO_2_ emission formation.

**Fig. 11:**
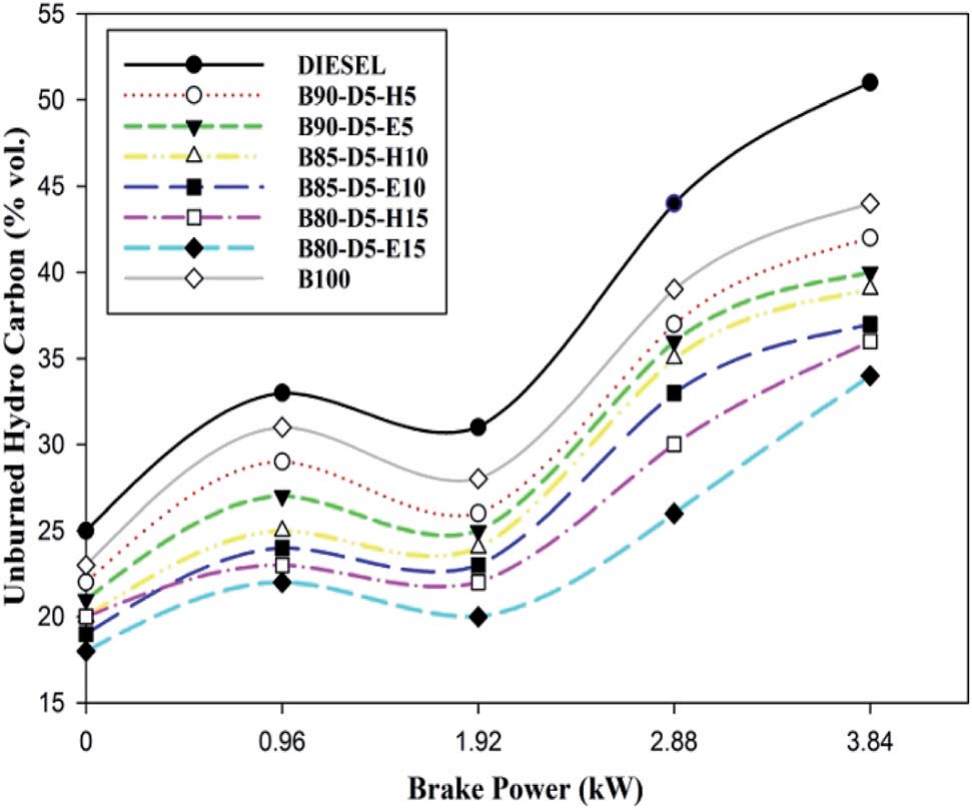
Variation of UBHC with brake power for diesel–biodiesel–alcohol blends.

The comparison of unburned hydrocarbon (UBHC) emission against brake power for biodiesel–alcohol blends is shown in Fig. 11. The UBHC is evidence of the quality of combustion. From Fig. 11, it was found that UBHC emissions rose with a rise in engine load. However, UBHC emission decreased for biodiesel–alcohol fuel blends compared with neat diesel. At all operating conditions, all tested fuel blends also showed lower UBHC emission compared with neat diesel. This is because excess oxygen content in the biodiesel blends improved the combustion process providing by proper atomization and evaporation. At 100% load, UBHC values of 51 ppm, 42 ppm, 36 ppm, 34 ppm, and 44 ppm were attained for conventional diesel, B90D5H5, B80-D5-H15, B80-D5-E15, and pure biodiesel, respectively. B80-D5-E15 has the minimum UBHC emission of 34 ppm, which is 33% lower than conventional diesel, 19% lower than B90-D5-H5 and 5.5% lower than B80-D5-H15. The presence of oxygen, a longer ID period, lower viscosity, and improved fuel–air mixture lead to proper combustion, resulting in lower UBHC emission for the biodiesel–alcohol fuel blends.

**Fig. 12:**
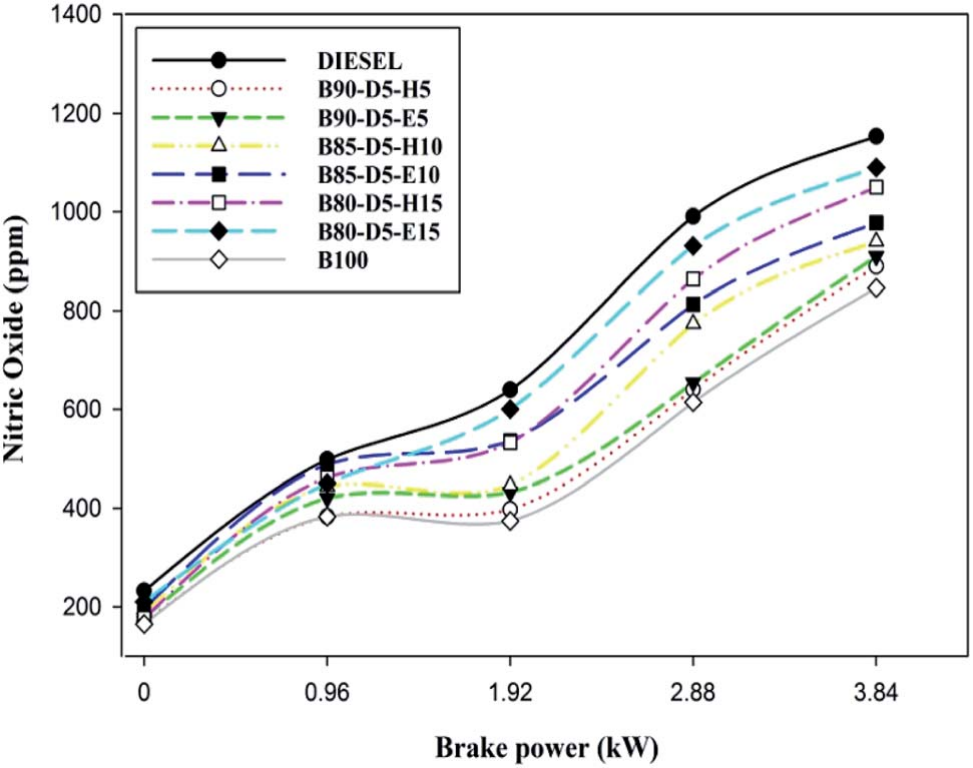
Variation of NO emission with brake power for diesel–biodiesel–alcohol blends.

**Fig. 13:**
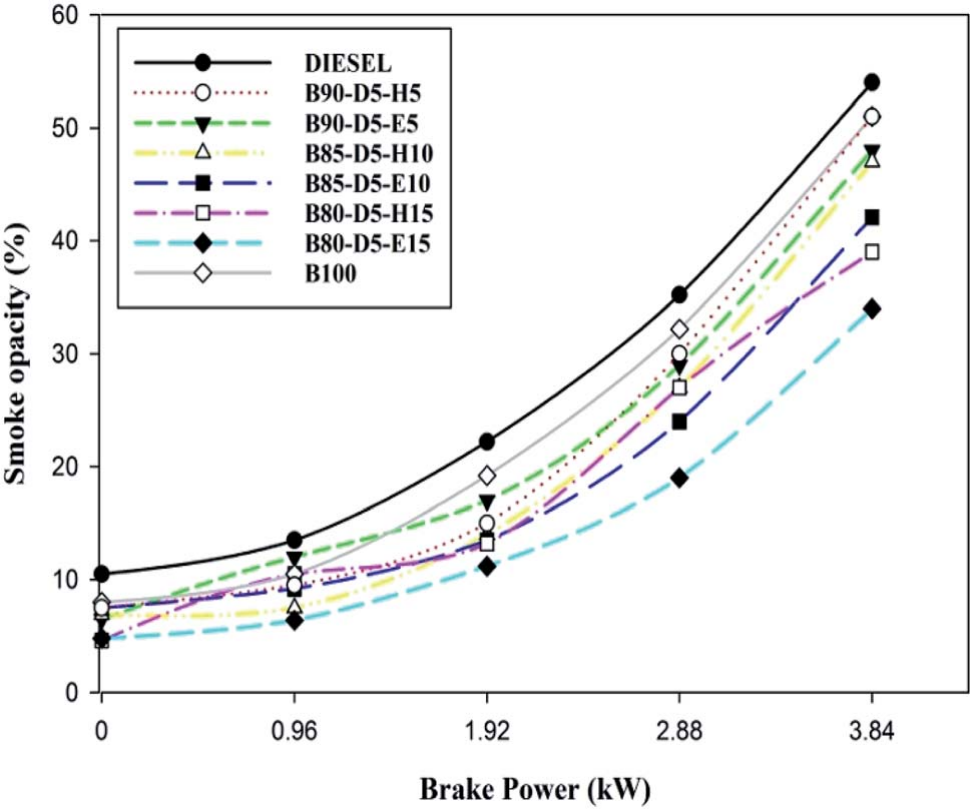
Variation of smoke opacity with brake power for diesel–biodiesel–alcohol blends.

Fig. 12 illustrates the variation of nitric oxide (NO) emission with BP for various biodiesel–alcohol fuel blends. The NO emission formation is mostly due to the presence of oxygen, occupancy time, and in-cylinder pressure and temperature. Also, NO formation mainly depends on the fuel amount burned at the premixed combustion zone compared to controlled and late combustion. Fig. 12 reveals that NO emission rose with a rise in engine load. However, the NO emission decreased when adding additional biodiesel in biodiesel–alcohol fuel blends. At all running conditions, biodiesel–diesel–hexanol blends showed lower NO emission compared with biodiesel–ethanol fuel blends. This is because adding extra hexanol in biodiesel–alcohol fuel blends raises the viscosity of the blends and increases their specific and latent heats, lowering the in-cylinder temperature and pressure which cause lower HRR with minimum NO emission formation. From Fig. 13, it was found that NO emissions of 1153 ppm, 890 ppm, 1050 ppm, 1090 ppm, and 846 ppm were attained in conventional diesel, B90-D5-H5, B80-D5-H15, B80-D5-E15, and pure biodiesel, respectively, at 100% load condition. Biodiesel has the minimum NO emission of 846 ppm, which is 26% less than conventional diesel, 5% less than B90-D5-H5, 19% less than B80-D5-H15, and 22% less than B80-D5-E15.

Fig. 13 illustrates the differences in smoke emissions against BP for biodiesel–diesel–alcohol blends. Smoke formation is mainly due to a rich air–fuel mixture, deficiency of oxygen, and lower cylinder temperature and pressure, burning velocity and latent heat of vaporization. Fig. 13 shows the smoke emission was reduced for biodiesel–alcohol fuel blends compared with conventional diesel, because oxygen availability and proper air–fuel mixture cause complete combustion resulting in lower smoke emission formation. B80-D5-E15 has the minimum smoke emission of 34%, which is 33% lower than pure biodiesel and 33% lower than B90-D5-H5. Biodiesel–diesel–ethanol blends have lower smoke emission compared with biodiesel–hexanol fuel blends. This is because of their lesser viscosity, lower latent heat of evaporation and faster combustion. At 100% load condition, the smoke opacity was higher for the tested fuels due to lower cylinder temperature and pressure and a higher quantity of fuels with a minimum ID period leading to incomplete combustion with maximum smoke opacity. Also, a rich fuel–air mixture is the reason for higher smoke at maximum load.

## 6. Conclusion

The influences of biodiesel–diesel–hexanol and biodiesel–diesel–ethanol blends on diesel engine performance, combustion and emission characteristics were experimentally examined. Also, this study examines biodiesel production from *Moringa oleifera* oil using sodium methoxide catalyst through a transesterification process. The main conclusions drawn from the research work are summarized below.

• The maximum biodiesel yield of 96.71% was obtained at the following reaction conditions: a molar ratio of 6 : 1, reaction temperature of 55 °C, catalyst of 1.8 wt%, reaction time of 120 min and a stirrer speed of 600 rpm.

• The maximum BTE of 28.8% was obtained in the B90-D5-H5 blend, which is 2.2% lower than conventional diesel, 4.2% higher than B80-D5-E15 and 4.4% higher than pure biodiesel. The higher viscosity of pure biodiesel reduced the evaporation rate and the lower heating value leads to lower BTE for pure biodiesel. The addition of 5% diesel in biodiesel blends improved the fuel properties.

• B90-D5-H5, conventional diesel, and B85-D5-H10 obtained HRR of 44 MJ per °CA, 76.5 MJ per °CA and 74 MJ per °CA, respectively. Biodiesel–diesel–hexanol blends have greater ignition delay and combustion duration compared to conventional diesel and biodiesel–diesel–ethanol blends.

• B80-D5-E15 has a minimum CO emission of 0.32% vol., which is 0.25% vol. lower than conventional diesel, 0.02% vol. lower than pure biodiesel and 0.20% vol. lower than B90-D5-H5. B80-D5-E15 has a maximum CO_2_ emission of 10.5% vol., which is 2.4% vol. higher than conventional diesel, similar to pure biodiesel and 1.2% vol. higher than B80-D5-E15. The B80-D5-E15 has the minimum UBHC emission of 34 ppm, which is 33% lower than conventional diesel, 19% lower than B90-D5-H5 and 5.5% lower than B80-D5-H15.

• Biodiesel has the minimum NO emission of 846 ppm, which is 26% lower than conventional diesel, 5% lower than B90-D5-H5, 19% lower than B80-D5-H15, and 22% lower than B80-D5-E15. B80-D5-E15, B80-D5-H15, and biodiesel have minimum smoke emissions of 34%, 39%, and 51%, respectively. Biodiesel–diesel–ethanol blends have lower smoke emission compared to biodiesel–diesel–hexanol blends.

• The higher and lower alcohols can be used as additives in biodiesel–diesel fuel. The engine was able to run up to 5% to 15% alcohol without any problem. From this research work, it is concluded that biodiesel–diesel and biodiesel–diesel-higher alcohol blends will be prominent alternative fuels for diesel engines.

## Nomenclature

ASTM: American society for testing and materials
aTDC: After top dead centre
B00: Diesel
B100: Biodiesel
B90-D5-H5: 90% biodiesel, 5% diesel, 5% hexanol
B90-D5-E5: 90% biodiesel, 5% diesel, 5% ethanol
D85-D5-H10: 85% diesel, 5% diesel, 10% hexanol
B85-D5-E10: 85% biodiesel, 5% diesel, 10% ethanol
B80-D5-H15: 80% biodiesel, 5% diesel, 15% hexanol
B80-D5-E15: 80% biodiesel, 5% diesel, 15% ethanol
BSEC: Brake specific energy consumption (MJ kW^−1^ h^−1^)
BSFC: Brake specific fuel consumption (kg kW^−1^ h^−1^)
BTE: Brake thermal efficiency (%)
CA: Crank angle
CD: Combustion duration (°CA)
CH_3_OH: Methanol
CH_3_ONa: Sodium methoxide
CO: Carbon monoxide (% vol.)
CO_2_: Carbon dioxide (% vol.)
CP: Cloud point
CPP: Cylinder peak pressure (bar)
HRR: Heat release rate (J/°CA)
ID: Ignition delay (°CA)
ME: Methyl ester
MOO: *Moringa oleifera* oil
NO: Nitric oxide (ppm)
NO_*X*_: Oxides of nitrogen (ppm)
UBHC: Unburned hydrocarbon (ppm)

## Conflicts of interest

There are no conflicts to declare.

## Supplementary Material
